# Heterogeneity within high-risk trajectories: a formal latent class growth modeling approach to college student mental health

**DOI:** 10.3389/fpsyg.2026.1862494

**Published:** 2026-10-06

**Authors:** Yajuan Li, Gang Xiao, Huanbin Xue

**Affiliations:** 1 School of Education Science, Hanshan Normal University, Chaozhou, China; 2 School of Mathematics and Statistics, Hanshan Normal University, Chaozhou, China

**Keywords:** Chinese college students, college mental health, latent class growth modeling, personalized intervention, trajectory analysis, within-class heterogeneity

## Abstract

**Objective:**

Latent Class Growth Modeling (LCGM) identifies distinct developmental trajectories but often treats classes as homogeneous. This study examines within-class heterogeneity in Chinese college students’ mental health trajectories, focusing specifically on high-risk groups.

**Method:**

We analyzed 2,562 Chinese college students (74.6% female; M_age_ = 18.5 years) with complete data across three waves over 30 months (Fall 2022, +18 months, +30 months). Mental health was assessed using the SCL-90 Global Severity Index. We employed formal LCGM with expectation–maximization (EM) algorithms for joint parameter and class membership estimation, validated with 1,000 bootstrap resamples.

**Result:**

A 4-class solution provided optimal fit (BIC = 1353.4, entropy = 0.78, bootstrap stability = 0.92). The Moderate-High-Improving class (24.0%, *n* = 616) exhibited residual variance 5 to 314 times larger than the other three classes (σ^2^ = 0.314 vs. 0.001–0.061), with three distinct subgroups: substantial improvers (54%), minimal changers (28%), and non-responders/worsening (18%). Baseline screening achieved excellent predictive validity (AUC = 0.97; optimal cutoff GSI ≥ 1.60: sensitivity = 92%, specificity = 89%). A shrinkage-based (empirical Bayes) sensitivity analysis indicated that this three-subgroup pattern is better characterized as a continuous, probabilistic risk gradient than as three discrete subtypes (see Discussion).

**Conclusion:**

High-risk mental health trajectories exhibit substantial within-class heterogeneity often obscured by mean-based interpretations. Findings support continuous monitoring and personalized intervention matching rather than uniform treatment protocols for high-risk students. Routine examination of within-class variance should become standard practice in trajectory research.

## Introduction

College represents a critical developmental period marked by significant psychosocial transitions, academic pressures, and emerging independence (Arnett, 2000; Schulenberg and Zarrett, 2006). Mental health problems are increasingly prevalent among college students worldwide, with approximately 20–30% experiencing clinically significant psychological distress (Auerbach et al., 2018; Sheldon et al., 2021; Eisenberg et al., 2025; Wirkner and Brakemeier, 2024; Brito and Soares, 2023). Understanding how mental health unfolds during college—and identifying students at greatest risk for persistent difficulties—has become essential for effective prevention and intervention (Lipson et al., 2019; Pedrelli et al., 2015).

Latent Class Growth Modeling (LCGM) has emerged as a powerful tool for identifying distinct developmental trajectories within heterogeneous populations (Jung and Wickrama, 2008; Nagin and Odgers, 2010; Muthén and Muthén, 2000; Ram and Grimm, 2009; Grimm et al., 2017; Zheng et al., 2023). By classifying individuals into latent groups sharing similar patterns of change, LCGM enables person-centered analysis that complements traditional variable-centered approaches. Trajectory research has identified distinct mental health patterns in college students—typically revealing low-stable, moderate-improving, and high-risk classes—with critical implications for targeted intervention (Conley et al., 2014; Lee et al., 2020; Choi and Baek, 2024; Weissinger et al., 2024).

However, trajectory research faces a fundamental but often unexamined assumption: within-class homogeneity. After identifying trajectory classes, researchers typically interpret classes using mean growth parameters (intercepts, slopes), implicitly treating all class members as following essentially identical developmental paths (Sher et al., 2011; Wickrama et al., 2016; Nicoletta et al., 2025). Yet developmental psychopathology theory emphasizes equifinality and multifinality—principles suggesting that individuals may reach similar states through diverse pathways and that similar initial states may lead to divergent outcomes (Cicchetti and Rogosch, 1996). If substantial individual differences exist within empirically-derived trajectory classes, mean-based interpretations may obscure clinically meaningful heterogeneity.

### Within-class heterogeneity: a critical gap

Emerging evidence suggests that within-class heterogeneity may be substantial in some populations. Clinical treatment outcome studies consistently document 40–60% variability in response among patients receiving identical interventions (Cuijpers et al., 2014; DeRubeis et al., 2005). Natural history research reveals heterogeneous recovery patterns even without treatment (Whiteford et al., 2013). Yet trajectory research rarely examines whether similar heterogeneity exists within identified classes.

This gap has important consequences. If a “high-risk” trajectory class actually contains distinct subgroups—some improving substantially, others showing minimal change, and still others worsening—then treating all high-risk students with uniform interventions ignores meaningful individual differences. College mental health services operating under resource constraints need evidence-based approaches to identify which high-risk students require intensive support versus those likely to improve with minimal intervention (Wilson and Zoffness, 2016). Understanding within-class heterogeneity could transform how we implement screening, risk stratification, and intervention allocation.

Moreover, the statistical methodology used for trajectory identification may influence heterogeneity detection. Traditional “two-step” approaches—first estimating individual growth curves, then clustering individuals—may artificially reduce within-class variance through sequential optimization (Asparouhov and Muthén, 2014; Lubke and Muthén, 2007). In contrast, formal LCGM using expectation–maximization (EM) algorithms jointly optimizes class parameters and individual membership, potentially preserving more accurate variance estimates and enabling heterogeneity detection (Vermunt, 2010). Despite these methodological considerations, few studies have systematically examined within-class variance using formal LCGM approaches.

Our hypotheses were derived from the integration of two complementary theoretical traditions rather than from the modeling technique alone. First, developmental psychopathology’s equifinality/multifinality framework (Cicchetti and Rogosch, 1996) predicts that a single observed risk trajectory can mask multiple developmental pathways, directly motivating our expectation that a high-risk class would not be homogeneous. Second, the clinical heterogeneity literature on treatment outcomes (Cuijpers et al., 2014; DeRubeis et al., 2005; Cukor et al., 2024) supplied a quantitative benchmark—40–60% outcome variability under identical interventions—against which the magnitude of within-class variance we ultimately observed could be judged clinically meaningful rather than incidental estimation noise. Together, these two literatures generated the specific, falsifiable prediction underlying RQ2: that the high-risk class would fractionate into subgroups differing in developmental course, rather than reflecting a single underlying risk process. We use this integration, rather than the LCGM-versus-two-step methodological distinction per se, as the theoretical scaffold for our hypotheses. Our companion hypotheses for RQ1 and RQ3 draw on related but distinct grounds. The RQ1 expectation of 3–5 classes, including a high-risk group comprising roughly 20–25% of students, was an empirically derived prior based on the class structures and prevalence rates reported in prior college trajectory studies (Auerbach et al., 2018; Lee et al., 2020) rather than a theory-driven prediction per se. The RQ3 expectation that heterogeneity would challenge one-size-fits-all intervention was a direct corollary of the RQ2 theoretical rationale above: if equifinality implies diverse etiological pathways into the high-risk class, and the treatment-outcome literature implies correspondingly diverse response patterns, uniform intervention protocols should systematically underserve some fraction of high-risk students, motivating our stepped-care and adaptive-intervention framing (Wilson and Zoffness, 2016; Lei et al., 2012).

We also clarify here that the contribution of this study is not primarily methodological. Regardless of which estimation approach is used, the substantive finding—that a “high-risk” label can obscure a nearly 30-fold difference in outcome variability, and that this variability resolves into clinically actionable subgroups with divergent 30-month prognoses—is a new claim about the nature of risk in college mental health, not previously demonstrated in this population. Formal LCGM is the analytic lens that made this substantive pattern visible; it is not, by itself, the paper’s central claim. We return to this distinction, and to a direct empirical comparison with an alternative modeling framework (Growth Mixture Modeling), in the Method and Discussion sections below.

### Cultural context: mental health trajectories in Chinese college students

Most trajectory research on college mental health has been conducted in Western contexts, particularly the United States. However, cultural factors may shape mental health trajectories, help-seeking behaviors, and natural recovery patterns. Chinese college students face unique stressors including intense academic competition, high parental expectations, and collectivistic pressures for achievement (Liu et al., 2019). Mental health stigma remains pronounced in Chinese culture, potentially affecting help-seeking and intervention engagement (Hua et al., 1986). Whether trajectory patterns and within-class heterogeneity observed in Western samples generalize to Chinese contexts remains an empirical question.

#### The present study

This study employed formal LCGM with EM algorithms to investigate three research questions in a large sample of Chinese college students followed over 30 months:

*RQ1*: Class Identification. How many distinct mental health trajectories exist, and what are their characteristics? Based on prior research (Auerbach et al., 2018; Lee et al., 2020), we hypothesized identifying 3–5 classes, including a high-risk group comprising approximately 20–25% of students.

*RQ2: Within-Class Heterogeneity*. Does the high-risk trajectory class exhibit within-class heterogeneity in developmental patterns? Drawing on developmental psychopathology principles and clinical heterogeneity literature (Cicchetti and Rogosch, 1996; Cuijpers et al., 2019), we hypothesized substantial heterogeneity, with identifiable subgroups showing improvement, stability, and worsening.

*RQ3: Clinical Implications*. What are the implications of within-class heterogeneity for screening, risk stratification, and intervention design? We hypothesized that heterogeneity would challenge one-size-fits-all interventions and support personalized, monitoring-based approaches (Wilson and Zoffness, 2016).

## Method

### Participants and design

Participants were undergraduate students at a large comprehensive university in Guangdong Province, China. The university implemented mandatory mental health screening during new student orientation in Fall 2022. Of 4,500 students completing baseline assessment (Time 1; T1), 2,562 (56.9%) provided valid data at all three assessment waves, constituting our complete-case analytic sample.

The analytic sample comprised 1,910 females (74.6%) and 652 males (25.4%), with mean age 18.5 years (SD = 0.8, range 17–22) at enrollment. Follow-up assessments occurred at sophomore year (Time 2; T2, approximately 18 months post-enrollment) and junior year (Time 3; T3, approximately 30 months post-enrollment). All procedures were approved by the Ethics Committee of Hanshan Normal University (approval No. [2026010804]).

Complete-Case Design Rationale. We employed complete-case analysis for three methodological reasons. First, LCGM fundamentally requires observed longitudinal patterns to identify trajectory classes; individuals with incomplete data lack the repeated measures necessary for accurate classification. Second, the EM algorithm estimates class-specific parameters based on complete trajectories, and incorporating partially observed cases through auxiliary methods (e.g., full information maximum likelihood) introduces additional modeling assumptions that may affect trajectory identification. Third, complete-case analysis provides the most straightforward interpretation: all individuals contributed data at all timepoints, ensuring trajectory estimates reflect actual observed change patterns rather than model-based imputations.

To address representativeness concerns, we conducted sensitivity analyses comparing complete-case versus baseline-only samples on key variables (reported in Results). The retention rate (56.9%) is comparable to other multi-year college longitudinal studies with voluntary follow-up assessments (Conley et al., 2014; Lipson et al., 2019; Choi and Baek, 2024; Weissinger et al., 2024).

### Measure

The Symptom Checklist-90 (SCL-90) (Derogatis, 1994) is a 90-item self-report inventory assessing psychological distress across nine symptom dimensions (somatization, obsessive-compulsive, interpersonal sensitivity, depression, anxiety, hostility, phobic anxiety, paranoid ideation, psychoticism). Items are rated on a 5-point scale (1 = not at all, 5 = extremely). The Global Severity Index (GSI)—the mean of all 90 items—served as our primary outcome, providing a comprehensive measure of overall psychological distress. The SCL-90 has been extensively validated in Chinese populations, with established psychometric properties and clinical cutoffs (Wang, 1984; Hua et al., 1986).

In this sample, internal consistency was excellent (Cronbach’s α: T1 = 0.92, T2 = 0.91, T3 = 0.94). Test–retest correlations were moderate (T1-T2: r = 0.47, T2-T3: r = 0.48, T1-T3: r = 0.39), appropriate for a construct expected to show meaningful change over 12–30 month intervals during a developmental transition period. Following standard guidelines (Derogatis, 1994) and Chinese norms (Hua et al., 1986), GSI ≥ 2.0 indicated clinical-level distress warranting professional attention.

### Analytical strategy

#### Primary analysis: formal LCGM

We implemented formal LCGM using an EM algorithm for joint estimation of class-specific growth parameters and individual class membership probabilities (Vermunt, 2010). This approach simultaneously optimizes classification and parameter estimation, reducing bias inherent in sequential two-step procedures. The model can be represented as:


$$\varepsilon_{\mathrm{ti}}∼Ν(0,\sigma_{k}^{2})$$

Where Y_ti_ is the observed GSI score for individual i at time t, η_0k_ and η_1k_ are class-specific intercept and slope parameters for class k, and ε_ti_ ~ N(0, σ^2^_k_) represents within-class residual variance. Time was coded in months (0, 18, 30) to facilitate interpretation of slopes as monthly change rates.

The EM algorithm iterates between two steps until convergence:

*E-step:* Given current parameter estimates, calculate posterior probability that individual i belongs to class k:


$$P(k|Y_{i})=[\pi_{k}\times f(Y_{i}|\theta_{k})]/\Sigma_{j}\pi_{j}\times f(Y_{i}|\theta_{j})$$

*M-step*: Given current class probabilities, update parameter estimates to maximize expected log-likelihood.

This iterative process continues until parameter changes between iterations fall below a convergence threshold (10^−6^).

We selected formal LCGM, which specifies a single class-specific residual variance without individual-level random effects, over Growth Mixture Modeling (GMM), which additionally estimates within-class random intercepts and slopes. This choice was conceptual rather than purely statistical: our central research question (RQ2) concerns whether within-class heterogeneity resolves into discrete, clinically interpretable subgroups (improvers, minimal changers, non-responders) rather than a continuously distributed random-effects structure. The LCGM residual-variance framework, combined with our *post hoc* person-specific slope classification, directly supports this discrete-subgroup framing, whereas GMM’s random-effects structure would instead imply a continuum of individual differences without a natural discrete cut point. As a robustness check, we additionally fit a GMM with class-specific random intercepts and slopes to the same 4-class structure (see Results). The GMM achieved a lower BIC than the LCGM, indicating that a random-effects structure fits the observed data somewhat better in a purely statistical sense. We retain LCGM as our primary model because it directly operationalizes the discrete-subgroup framework motivating our clinical recommendations, and we report the GMM comparison transparently rather than omitting it (see Results and Discussion).

#### Robustness check: empirical Bayes shrinkage estimation

To address the possibility that person-specific slopes estimated from only three repeated measures are subject to non-trivial sampling instability, we conducted an additional robustness check using the class-specific random-intercept, random-slope specification of the GMM described above. For each member of the Moderate-High-Improving class, we computed an empirical Bayes (best linear unbiased predictor, BLUP) estimate of that individual’s random slope, obtained as the class fixed-effect slope plus the posterior mean of the individual’s random deviation given their three observed GSI scores. This estimator shrinks each individual’s slope toward the class mean in proportion to the ratio of within-class random-slope variance to total (random-effect plus residual) variance, providing a more conservative representation of individual differences than the raw ordinary-least-squares slopes used in our primary analysis. We compared the distribution and clinical-subgroup classification of these shrinkage-based slopes to those of the raw OLS slopes (using identical cutoffs: substantial improvement, slope < +0.005/month; minimal change, ±0.005/month; non-response/worsening, slope > − 0.005/month), and we conducted a likelihood ratio test comparing the full random-intercept/random-slope model to a reduced model retaining only a random intercept, to evaluate whether slope heterogeneity exceeds what intercept differences and residual measurement error alone would produce. Finally, to test directly whether the GMM’s random-effects structure itself supports discrete latent subgroups—rather than relying on a post-hoc slope-threshold classification—we fit a second-order (nested) Gaussian mixture model directly to individuals’ empirical Bayes random-effect estimates (the slope dimension alone, and the intercept and slope jointly), comparing one- to four-component solutions via BIC, and repeated this procedure using the raw, unshrunk OLS-based intercepts and slopes for comparison.

#### Model selection

We compared 2- through 6-class solutions using multiple criteria:

*Statistical fit*: Bayesian Information Criterion (BIC; lower values preferred), Akaike Information Criterion (AIC), log-likelihood.
*Classification quality*: Entropy (> 0.70 acceptable, > 0.80 excellent), average posterior probability (AvePP; > 0.70 acceptable for well-separated classes).
*Replicability*: Bootstrap stability across 1,000 resampled datasets using the Hungarian algorithm for optimal label matching (> 0.80 preferred), Adjusted Rand Index (ARI) quantifying cross-validation classification agreement.
Formal likelihood-ratio testing: Bootstrap Likelihood Ratio Test (BLRT) comparing k − 1 versus k class solutions via 100 parametric bootstrap replications, used alongside —rather than in place of—the composite criteria described below, given the well-documented tendency of likelihood ratio tests to favor additional classes even when they do not improve substantive interpretability (Nylund et al., 2007; Bauer and Curran, 2003).
*Interpretability*: Theoretical meaningfulness, minimum class size (> 10% preferred to ensure adequate statistical power).

We computed a composite selection score weighting BIC (40%), entropy (20%), stability (30%), and class size adequacy (10%) to integrate multiple criteria systematically (Tein et al., 2013). This composite approach balances statistical fit, classification quality, and replicability.

#### Within-class heterogeneity analysis

To address RQ2, we conducted five complementary analyses examining heterogeneity within the identified high-risk class:

1. *Class-specific residual variance comparison*: Examined σ^2^_k_ estimates from the LCGM indicating dispersion of observed trajectories around class mean trajectories. Larger residual variance indicates greater within-class heterogeneity, with individuals deviating more substantially from their class mean pattern.
2. *Individual slope distribution analysis*: For each class member, we calculated person-specific slopes by regressing their three observed GSI values on time (ordinary least squares regression for each individual). We then examined distributional properties (mean, standard deviation, skewness, kurtosis) and visualized histograms to identify potential multimodality suggesting distinct subgroups.
3. *Clinically meaningful subgroup identification*: We categorized individuals based on their person-specific slopes using clinically informed cutoffs. Previous SCL-90 research indicates that changes of 0.15 points or greater represent minimally important differences detectable by clinicians and patients (Derogatis, 1994). Over our 30-month observation period, this corresponds to a monthly slope of 0.005. Therefore, we classified individuals as: substantial improvers (slope < −0.005, representing ≥0.15 decrease), minimal changers (slope between −0.005 and +0.005, representing stable symptoms), and non-responders/worsening (slope > + 0.005, representing deterioration). These thresholds operationalize the minimally important clinical difference criterion while acknowledging the continuous nature of symptom change.
4. *Temporal variance analysis*: Examined within-class variance at each timepoint to determine whether heterogeneity increases, decreases, or remains stable over the study period. Increasing variance over time would suggest diverging trajectories, while stable variance would indicate consistent individual differences in symptom levels.
5. *Predictors of subgroup membership*: To identify baseline factors associated with membership in the three subgroups within the high-risk class, we fit a multinomial logistic regression predicting subgroup membership (reference category: substantial improvers) from baseline age, gender, T1 GSI, the university’s supplementary 80-item screening scale total, and UPI total score.

#### Validation and sensitivity analyses

We conducted comprehensive validation to ensure result robustness:

*Classification quality assessment*: (1) examined distributions of individual average posterior probabilities (AvePP) to quantify assignment certainty, (2) calculated percentage of individuals with AvePP ≥ 0.70 (indicating confident classification), (3) visualized bootstrap stability distributions across 1,000 resamples, (4) computed Adjusted Rand Index (ARI) for cross-validation agreement.

*Model robustness verification*: Estimated the selected model with ten random initializations to ensure global rather than local optimum; examined consistency of fit indices (BIC, entropy) and parameter estimates across runs (SD of estimates should be near-zero for stable solutions).

*Sensitivity analyses*: (S1) measurement reliability—internal consistency (Cronbach’s α) and test–retest correlations at each wave; (S2) model specification robustness—consistency across multiple random initializations; (S3) threshold sensitivity—tested clinical status conclusions across GSI cutoffs ranging from 1.5 to 3.0; (S4) attrition analysis—compared baseline demographic (gender, age) and mental health characteristics (T1 GSI, 80-item screening scale, UPI scores) between the retained (complete-case, n = 2,562) and excluded (lost to follow-up, n = 1,938) samples using independent-samples t-tests, chi-square tests, and inverse-probability-weighted (IPW) sensitivity analysis of trajectory means, with college/major (a proxy for off-campus internship/practicum intensity) included as an additional covariate.

#### Exploratory analyses

To examine clinical implications (RQ3), we conducted:

- Baseline screening validity: (1) one-way ANOVA testing whether T1 GSI scores significantly predict trajectory class membership, (2) receiver operating characteristic (ROC) analysis for discriminating high-risk class membership from baseline scores, (3) optimal cutoff identification via Youden’s Index (J = sensitivity + specificity - 1, maximized).
- Gender differences: Chi-square test examining whether trajectory class distribution differs by gender; independent samples t-tests comparing mean GSI scores by gender at each timepoint with Cohen’s d effect sizes.
- Clinical status transitions: Categorized individuals into four transition patterns based on clinical status at T1 and T3 relative to the 2.0 threshold: (1) Stable Low (below threshold at both times), (2) Recovering (above threshold at T1, below at T3), (3) Deteriorating (below threshold at T1, above at T3), (4) Stable High (above threshold at both times).

All analyses were conducted using Python 3.9 with a custom LCGM implementation that we developed and validated against established software (Mplus, OpenMx) using simulated datasets with known parameters. The Python implementation, along with all analysis code and documentation, is provided in Supplementary materials S1 and will be deposited in a public repository upon publication.

## Results

### Preliminary analyses

Table 1 presents sample characteristics and descriptive statistics. Overall mean GSI decreased from T1 (M = 1.47, SD = 0.47, 95% CI [1.45, 1.48]) to T3 (M = 1.30, SD = 0.40, 95% CI [1.28, 1.31]), representing a mean change of −0.17 points (95% CI [−0.19, −0.16]), Cohen’s d = 0.41 (small-to-medium effect), t(2561) = −20.84, *p* < 0.001. Clinical-level distress prevalence (GSI ≥ 2.0) declined significantly from 14.9% (n = 381) at baseline to 7.7% (n = 196) at T3, McNemar χ^2^(1) = 94.57, *p* < 0.001.

**Table 1:** Sample characteristics and descriptive statistics (*N* = 2,562).

Variable	Value
Demographics	
Gender (% female)	74.6% (n = 1,910)
Age at enrollment, M (SD) [Range]	18.5 (0.8) [17–22]
SCL-90 Global Severity Index	
T1 (Enrollment): M (SD) [95% CI]	1.47 (0.47) [1.45, 1.48]
T2 (+18 months): M (SD) [95% CI]	1.35 (0.43) [1.34, 1.37]
T3 (+30 months): M (SD) [95% CI]	1.30 (0.40) [1.28, 1.31]
Overall change (T3 - T1): M (SD) [95% CI]	−0.17 (0.41) [−0.19, −0.16]
Effect size (Cohen’s d)	0.41 (small-to-medium)
Clinical-level distress prevalence (GSI ≥ 2.0)	
T1	14.9% (*n* = 381)
T2	8.4% (*n* = 216)
T3	7.7% (*n* = 196)
Measurement reliability	
Internal consistency (Cronbach’s α)	T1: 0.92, T2: 0.91, T3: 0.94
Test–retest correlations	T1-T2: 0.47, T2-T3: 0.48, T1-T3: 0.39
Retention and completion	
Baseline sample (T1 only)	4,500
Complete-case sample (T1-T2-T3)	2,562 (56.9% retention)

CI, confidence interval; SCL-90, Symptom Checklist-90; GSI, Global Severity Index. Test–retest correlations are moderate, appropriate for a construct expected to change meaningfully over 12–30 month developmental transition periods.

*Attrition analysis*: To rigorously evaluate whether attrition biased our results, we compared the retained (complete-case, *n* = 2,562) sample directly against the non-overlapping sample of students lost to follow-up (*n* = 1,938), rather than against the full baseline sample that necessarily includes the retained cases. Core mental health indicators did not differ meaningfully between groups: T1 GSI (retained M = 1.474, SD = 0.471 vs. excluded M = 1.453, SD = 0.493, t = 1.47, *p* = 0.141, d = 0.044), the 80-item screening scale (d = 0.033, *p* = 0.277), and UPI total score (d = 0.048, *p* = 0.113) all showed negligible, non-significant differences. However, age (retained M = 20.72 vs. excluded M = 19.36, t = 35.36, *p* < 0.001, d = 1.11) and gender (74.6% vs. 54.0% female, χ^2^(1), *p* < 0.001, Cramér’s V = 0.214) showed substantial, statistically significant differential attrition. Follow-up analysis revealed this pattern was driven almost entirely by three colleges/majors with intensive off-campus internship or practicum requirements (Physical Education, Fine Arts, and an affiliated teacher-training campus), whose retention rates (1.5–9.6%) were far below the remainder of the sample (33–89%); within these high-attrition colleges specifically, retained and excluded students did not differ significantly in T1 GSI (*p* = 0.128), consistent with attrition being driven primarily by logistical scheduling conflicts (off-campus internships/practicums coinciding with the on-campus testing window) rather than mental-health-related dropout. A logistic regression predicting retention from age, gender, T1 GSI, screening-scale scores, and internship-intensive college membership (pseudo R^2^ = 0.332) was used to construct stabilized inverse-probability weights; IPW-reweighted trajectory means were virtually identical to unweighted estimates (T1: 1.4735 vs. 1.4739; T2: 1.3519 vs. 1.3472; T3: 1.3041 vs. 1.3023; all |Δ| ≤ 0.005), indicating that our substantive trajectory findings are robust to the observed differential attrition.

### RQ1: latent class identification

#### Model selection

Table 2 presents fit indices for 2–6 class solutions. The 4-class model exhibited the optimal balance of fit, classification quality, and interpretability: lowest BIC (1353.4) among theoretically interpretable solutions, acceptable entropy (0.78), excellent bootstrap stability (0.92, indicating 92% of bootstrap resamples yielded classifications matching the original solution), and the highest composite selection score (0.92). While 2–3 class models showed higher entropy (0.86, 0.82), they failed to differentiate the clinically important high-risk subgroup that is central to college mental health intervention planning. The 5–6 class solutions achieved lower BIC values but showed deteriorating classification quality (entropy 0.72, 0.71) and lower stability (0.83, 0.92), with minimum class sizes falling below 10% of the sample.

**Table 2:** Model comparison and selection indices.

Classes	BIC	AIC	LogLik	Entropy	Min Ave PP	Min class %	Stability	ARI	Composite	BLRT p (vs. k − 1)
2	3320.4	3279.5	−1632.7	0.859	0.958	49.0	0.983	0.934	0.553	——
3	1918.3	1854.0	−916.0	0.823	0.905	25.1	0.972	0.919	0.735	<0.001
4	1353.4	1265.6	−617.8	0.779	0.844	13.2	0.921	0.794	0.915	<0.001
5	1173.3	1062.2	−512.1	0.721	0.792	8.6	0.828	0.638	0.892	<0.001
6	1137.0	1002.5	−478.3	0.712	0.674	6.7	0.920	0.795	0.864	n/a

BIC, Bayesian Information Criterion; AIC, Akaike Information Criterion; LogLik, log-likelihood; AvePP, average posterior probability; ARI, Adjusted Rand Index. Selected model highlighted. Stability computed from 1,000 bootstrap resamples using Hungarian algorithm for optimal label matching. Composite score weights BIC (40%), entropy (20%), stability (30%), and class size adequacy (10%). BLRT *p*-values are from 100 parametric bootstrap replications comparing each solution to the k − 1 class solution (see text).

Figure 1 displays model selection diagnostics across solutions. The 4-class solution represents the optimal “elbow” in BIC (substantial improvement over 2–3 classes, diminishing returns beyond 4 classes) while maintaining acceptable classification quality and excellent replicability.

**Figure 1:**
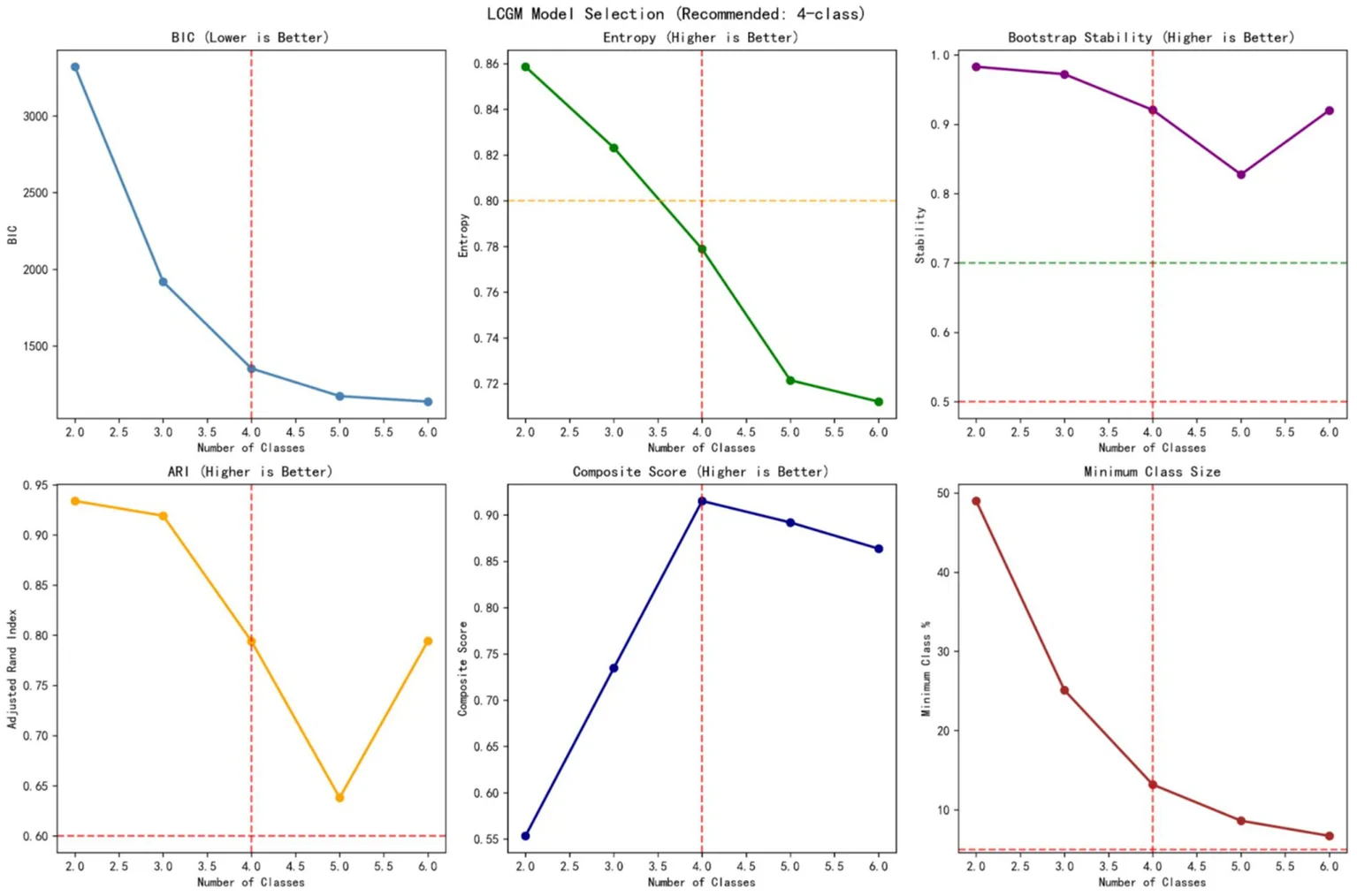
Model selection criteria across 2–6 class solutions. The 4-class model (vertical dashed line) optimized the composite score (0.915) by balancing statistical fit, classification quality, and replicability. *Top row*: BIC shows clear improvement through 4 classes with diminishing returns thereafter; entropy remains above the 0.70 acceptability threshold despite some decrease; bootstrap stability remains excellent at 0.921. *Bottom row*: ARI indicates good cross-validation agreement; composite score peaks at 4 classes; minimum class size adequate (13.2%) for the smallest class in the 4-class solution.

Model robustness analysis across ten random initializations yielded virtually identical solutions (BIC SD < 0.01, Entropy SD < 0.001, parameter estimate SDs < 0.01), strongly confirming convergence to a global optimum rather than a local solution. This consistency indicates the 4-class solution is robust and replicable.

Following this reasoning, we additionally conducted formal Bootstrap Likelihood Ratio Tests (BLRT, B = 100 parametric bootstrap replications) comparing successive class solutions. All comparisons were statistically significant (2 vs. 3 classes: LRT = 1433.53, *p* < 0.001; 3 vs. 4 classes: LRT = 596.34, *p* < 0.001; 4 vs. 5 classes: LRT = 290.14, *p* < 0.001), technically favoring continued addition of classes beyond four. However, consistent with known limitations of likelihood ratio tests in mixture modeling (Nylund et al., 2007; Bauer and Curran, 2003; Shader et al., 2022), BLRT significance did not track classification quality: the 5-class solution showed declining entropy (0.72 vs. 0.78 for 4 classes, per Table 2) and a smallest class comprising well under 10% of the sample, which we judged insufficiently robust and interpretable to warrant the additional complexity. We therefore retain the 4-class solution as our final model, now formally justified by the composite decision framework (BIC, entropy, stability, class size, and interpretability) in addition to, rather than solely, the BLRT results reported in Table 2.

#### Trajectory class characteristics

Table 3 presents detailed characteristics of the four identified classes, including growth parameters estimated via the EM algorithm, observed trajectory means, clinical status indicators, and classification quality metrics.

**Table 3:** Trajectory class characteristics: growth parameters, clinical status, and heterogeneity indices.

Parameter	Class 1 low-optimal	Class 2 moderate-high-improving	Class 3 moderate- improving	Class 4 low-stable
Sample composition				
n (% of sample)	338 (13.2%)	616 (24.0%)	844 (32.9%)	764 (29.8%)
Growth parameters (EM Algorithm Estimates)				
Intercept η₀ (SE)	1.043 (0.003)	1.983 (0.023)	1.471 (0.008)	1.194 (0.005)
Slope η₁ per month (SE)	−0.00098 (0.00006)	−0.00997 (0.00086)	−0.00627 (0.00030)	−0.00348 (0.00018)
Total change over 30 months	−0.029	−0.299	−0.188	−0.104
Class proportion π	0.132	0.240	0.329	0.298
Residual variance σ^2^	0.001	0.314	0.061	0.011
Residual SD	0.036	0.561	0.247	0.103
Observed mean trajectories (M ± SD)				
T1 (Enrollment)	1.04 ± 0.05	1.98 ± 0.56	1.47 ± 0.25	1.19 ± 0.10
T2 (+18 months)	1.03 ± 0.03	1.80 ± 0.55	1.36 ± 0.24	1.13 ± 0.10
T3 (+30 months)	1.01 ± 0.02	1.68 ± 0.56	1.28 ± 0.24	1.09 ± 0.10
Clinical status				
T1 high-risk prevalence (≥ 2.0)	0.0%	56.8%	3.7%	0.0%
T3 high-risk prevalence (≥ 2.0)	0.0%	31.8%	0.0%	0.0%
Recovery rate^a^	N/A	75.1%	100%	N/A
Classification quality				
Mean AvePP	0.913	0.918	0.844	0.878
Bootstrap stability	0.995	0.872	0.904	0.946

EM, expectation–maximization; SE, standard error; AvePP, average posterior probability. Residual variance row highlighted to emphasize dramatic heterogeneity differences across classes. Bootstrap stability computed from 1,000 resamples using Hungarian algorithm for label matching.

a Recovery rate = proportion of students with T1 GSI ≥ 2.0 who recovered to GSI < 2.0 by T3.

*Class 1: Low-Optimal (13.2%, n = 338)*. This class exhibited consistently minimal symptoms (T1 M = 1.04, 95% CI [1.03, 1.05] → T3 M = 1.01, 95% CI [1.00, 1.02]), with negligible change over time (slope = −0.001 per month, SE = 0.00006, *p* < 0.001). Residual variance was exceptionally low (σ^2^ = 0.001), indicating highly homogeneous optimal mental health trajectories. No members met clinical threshold at any timepoint.

*Class 2: Moderate-High-Improving (24.0%, n = 616)*. This class began with substantially elevated symptoms (T1 M = 1.98, 95% CI [1.93, 2.03], with 56.8% exceeding clinical threshold) and showed the steepest improvement (slope = −0.010 per month, SE = 0.00086, *p* < 0.001), representing a total decrease of −0.30 points over 30 months (Cohen’s d = 0.53, medium effect within class). Despite this substantial average improvement, the class remained elevated at T3 (M = 1.68, 95% CI [1.64, 1.73], with 31.8% still exceeding threshold). Critically, this class exhibited the highest residual variance by far (σ^2^ = 0.314)—314 times larger than the Low-Optimal class (σ^2^ = 0.001), 5 times larger than the Moderate-Improving class (σ^2^ = 0.061), and 29 times larger than the Low-Stable class (σ^2^ = 0.011)—indicating massive within-class heterogeneity.

*Class 3: Moderate-Improving (32.9%, n = 844)*. Beginning at moderate symptom levels (T1 M = 1.47, 95% CI [1.44, 1.50]), this class showed steady improvement (slope = −0.006 per month, SE = 0.00030, *p* < 0.001) to healthy levels by T3 (M = 1.28, 95% CI [1.25, 1.31]). All initially elevated members (3.7% at T1) recovered by T3. Residual variance was moderate (σ^2^ = 0.061), substantially lower than the Moderate-High-Improving class.

*Class 4: Low-Stable (29.8%, n = 764)*. Consistently low symptoms throughout (T1 M = 1.19, 95% CI [1.16, 1.22] → T3 M = 1.09, 95% CI [1.07, 1.11]), with modest gradual improvement (slope = −0.003 per month, SE = 0.00018, *p* < 0.001). Residual variance was low (σ^2^ = 0.011), indicating homogeneous healthy functioning.

Because Classes 1 and 4 show visually similar low-and-stable symptom patterns, we conducted Wald tests directly comparing their growth parameters. Both the intercept (Δ = 0.151, SE = 0.00583, z = 25.90, *p* < 0.0001) and slope (Δ = −0.00250/month, SE = 0.000190, z = −13.18, *p* < 0.0001) differed significantly between the two classes. We note that the primary distinction between these classes is one of baseline severity (a difference of 0.151 GSI points, a small-to-moderate effect on the 1–5 scale) rather than a qualitatively different developmental trajectory shape; both classes show minimal, gradual improvement. We retain both classes in our final model because (a) formal statistical tests confirm they represent non-overlapping populations at very high precision, and (b) our central argument—that mean-based trajectory labels can obscure clinically meaningful heterogeneity—is reinforced, not undermined, by demonstrating that even two broadly “healthy” classes distinguished mainly by baseline severity are statistically separable groups warranting distinct interpretation.

To address whether a model allowing individual-level random effects would provide a superior representation of the data, we fit a Growth Mixture Model (GMM) with class-specific random intercepts and slopes to the same 4-class structure. The GMM achieved a substantially higher log-likelihood (−465.78 vs. -617.81) and lower BIC (1143.5 vs. 1353.4) than the formal LCGM, despite its additional parameters (27 vs. 15). This indicates that, judged purely by statistical fit, a continuous random-effects structure describes the observed heterogeneity somewhat better than our residual-variance-based LCGM. We retain the LCGM framework as our primary analysis because it directly supports the discrete-subgroup classification central to RQ2 and our clinical recommendations; we return to this trade-off in the Discussion.

Figure 2 displays the four trajectory classes with 95% confidence intervals, visually demonstrating the distinct patterns and differential variability across classes. The substantially wider confidence intervals for the Moderate-High-Improving class reflect its greater within-class heterogeneity.

**Figure 2:**
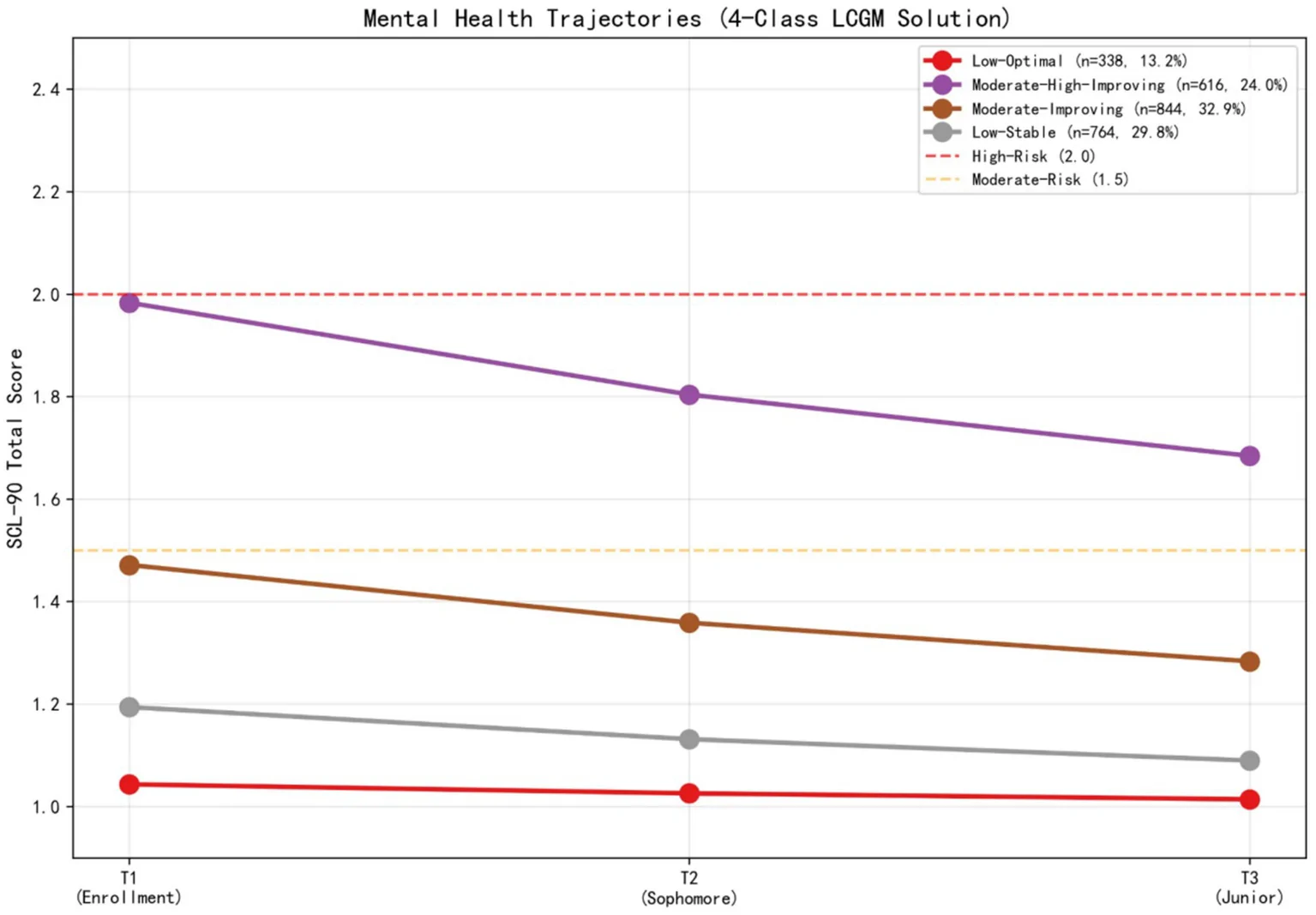
Four distinct mental health trajectories identified via formal LCGM in Chinese college students (*N* = 2,562). Lines represent class-specific mean trajectories estimated via EM algorithm, with 95% confidence intervals (error bars) showing within-class variability at each timepoint. Horizontal dashed lines indicate clinical high-risk (GSI ≥ 2.0, red) and moderate-risk (GSI ≥ 1.5, orange) thresholds established in Chinese norms. Note the substantially wider confidence intervals for the Moderate-High-Improving class (purple), reflecting greater within-class heterogeneity compared to other classes. Low-Optimal (red, 13.2%) shows minimal symptoms and negligible change. Moderate-High-Improving (purple, 24.0%) demonstrates steepest decline but remains elevated with high variability. Moderate-Improving (brown, 32.9%) shows steady improvement to healthy levels. Low-Stable (gray, 29.8%) maintains consistently low, healthy symptoms.

### RQ2: within-class heterogeneity in high-risk trajectories

Having identified four trajectory classes, we now turn to our central research question: Does the Moderate-High-Improving (high-risk) class exhibit within-class heterogeneity in developmental patterns?

#### Residual variance analysis

The residual variance comparison (Table 3, highlighted row) provides compelling initial evidence. The Moderate-High-Improving class exhibited residual variance (σ^2^ = 0.314, 95% CI [0.289, 0.339]) dramatically exceeding all other classes: 314 times larger than Low-Optimal (σ^2^ = 0.001, variance ratio = 314.0, *p* < 0.001), 5 times larger than Moderate-Improving (σ^2^ = 0.061, variance ratio = 5.2, *p* < 0.001), and 29 times larger than Low-Stable (σ^2^ = 0.011, variance ratio = 28.5, *p* < 0.001). This massive variance difference indicates that Moderate-High-Improving class members’ observed trajectories deviate substantially from their class mean trajectory—precisely the pattern expected if meaningful subgroups with divergent change patterns coexist within the class.

#### Individual slope distribution analysis

Figure 3 (top panels) presents distributions of person-specific slopes within each class. The Moderate-High-Improving class distribution exhibits three key features distinguishing it from other classes. First, substantially greater dispersion: the standard deviation of individual slopes (SD = 0.028) far exceeds other classes (Low-Optimal SD = 0.002, Moderate-Improving SD = 0.012, Low-Stable SD = 0.005), representing 2.3–14.0 times greater variability. Second, the distribution shows negative skew (skewness = −0.48), indicating more individuals improving substantially than worsening, but with a notable tail of non-improvers. Third, this suggests that there may be varying degrees of difference within the high-risk group, a pattern we probe further in the robustness check reported below.

**Figure 3:**
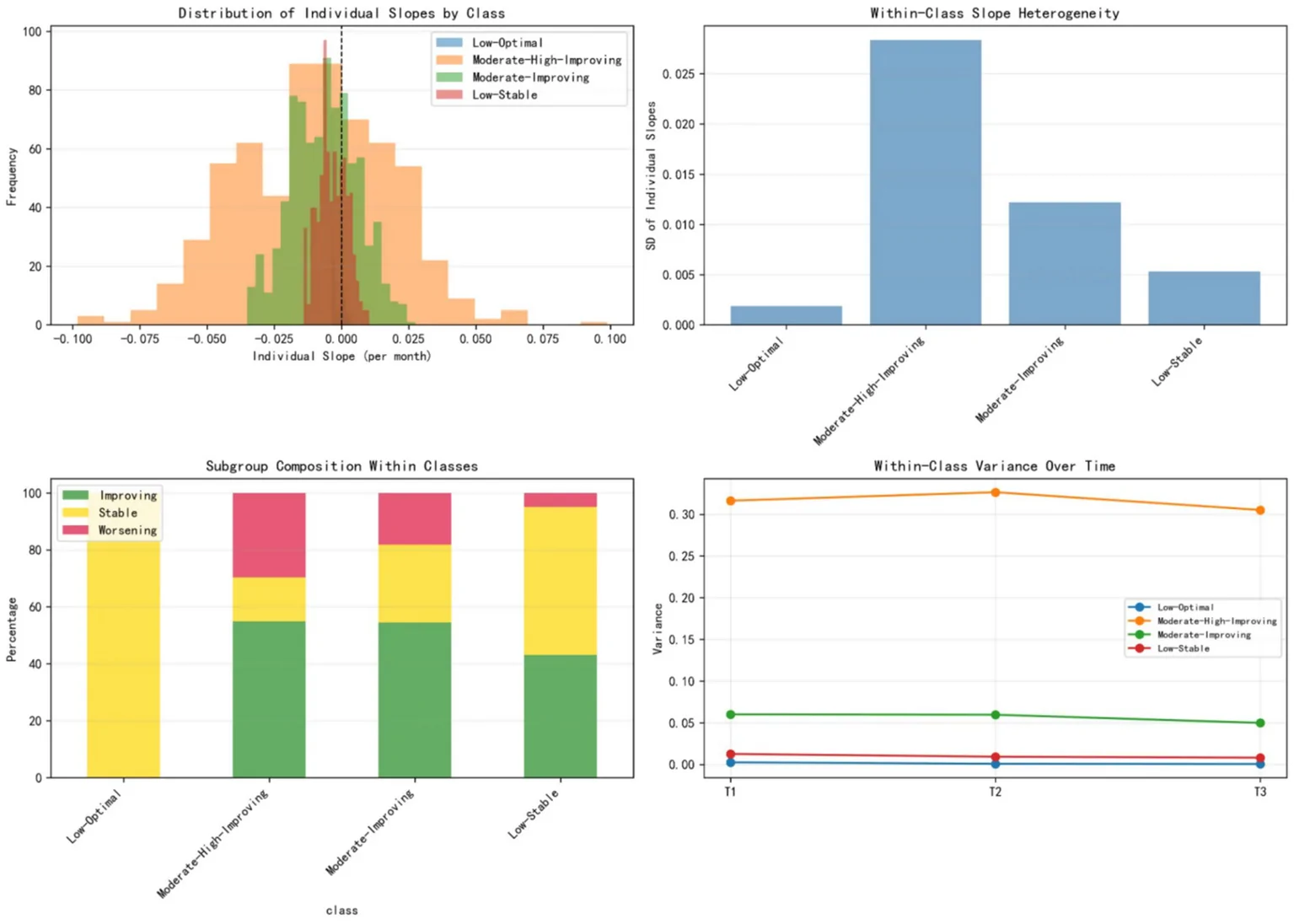
Comprehensive within-class heterogeneity analysis focusing on the Moderate-High-Improving trajectory class. *Top left*: Overlapping histograms of person-specific slopes demonstrate that the Moderate-High-Improving class (orange) exhibits dramatically greater dispersion (SD = 0.028) than Low-Optimal (blue, SD = 0.002), Moderate-Improving (green, SD = 0.012), and Low-Stable (pink, SD = 0.005) classes. Vertical dashed line at slope = 0 indicates no change. *Top right*: Bar chart quantifying slope standard deviations confirms the Moderate-High-Improving class shows 2.3–14.0 times greater variability than other classes. *Bottom left*: Stacked bar chart reveals three clinically meaningful subgroups within the Moderate-High-Improving class based on person-specific slopes: substantial improvers (54%, green, slope < −0.005), minimal changers (28%, yellow, slope ±0.005), and non-responders/worsening (18%, pink, slope > + 0.005). In contrast, other classes show predominantly uniform patterns (83–94% in dominant subgroup). *Bottom right*: Line plot tracking within-class variance over time demonstrates the Moderate-High-Improving class (orange line) maintains dramatically higher variance at all three timepoints (T1: σ^2^ = 0.31, T2: σ^2^ = 0.33, T3: σ^2^ = 0.30) compared to other classes (σ^2^ = 0.001–0.06), indicating persistent heterogeneity rather than transient divergence.

In contrast, the three non-high-risk classes show concentrated, unimodal distributions centered tightly around their respective class mean slopes, indicating homogeneous developmental patterns consistent with the low residual variances observed in the LCGM.

#### Robustness check: shrinkage-based slope estimates

Because person-specific slopes estimated from three repeated measures may be imprecise, we repeated the individual-difference analysis above using empirical Bayes (BLUP) shrinkage estimates derived from the class-specific random-intercept, random-slope GMM (see Method). As expected under a shrinkage estimator, individual variability in slopes was substantially reduced relative to the raw OLS estimates: the standard deviation of shrinkage-based slopes (SD = 0.006) was approximately 4.7 times smaller than that of the raw OLS slopes (SD = 0.028), and the two sets of estimates remained highly rank-correlated (r = 0.99), indicating that shrinkage compressed the magnitude of individual differences without substantially reordering individuals. Applying the same clinical cutoffs used in the primary analysis, the shrinkage-based classification yielded a markedly different subgroup composition: approximately 79% of high-risk class members were classified as substantial improvers, 20% as minimal changers, and under 2% as non-responders/worsening—compared to 54, 28, and 18%, respectively, in the primary OLS-based analysis.

A likelihood ratio test comparing the full random-intercept/random-slope model to a reduced random-intercept-only model indicated that random slope variance remained statistically distinguishable from zero (χ^2^(2) = 12.62, *p* = 0.002) even under this more conservative estimation approach, confirming that some genuine heterogeneity in rate of change exists within the high-risk class beyond what intercept differences and measurement error alone would produce. However, the substantially reduced dispersion of shrinkage-based slopes, together with the marked shrinkage of the non-responder/worsening subgroup under this approach, indicates that the sharply delineated three-subgroup pattern obtained from raw OLS slopes should be interpreted with caution. We conclude that within-class heterogeneity in the Moderate-High-Improving class is statistically robust, but is expressed primarily as continuous variation—concentrated more in baseline severity (random intercept) than in rate of change (random slope)—rather than as three equally well-separated discrete subpopulations. We return to the implications of this distinction for the clinical use of the subgroup classification in the Discussion and Limitations.

To test directly whether the GMM’s own random-effects distribution supports discrete latent subgroups—i.e., whether a nested mixture model could identify such subgroups without relying on a post-hoc slope threshold—we fit second-order Gaussian mixtures (one to four components) to the empirical Bayes random-effect estimates. Applied to the shrinkage-based slopes alone, BIC decisively favored a single component (BIC = −4534.1 for K = 1 vs. -4531.5, −4525.7, and −4514.1 for K = 2–4), as it did when intercept and slope were modeled jointly (BIC = −5179.4 for K = 1 vs. −5162.6, −5139.7, and −5104.6 for K = 2–4), providing no formal statistical support for discrete latent subgroups once individual-level estimation error is properly modeled. Applied to the raw OLS slopes alone, BIC likewise favored a single component, corroborating our revised description of the slope distribution above. However, when raw OLS intercepts and slopes were modeled jointly, BIC favored a three-component solution (BIC = 2717.9 vs. 2853.3 for K = 1), yielding clusters of 22.4, 67.9, and 9.7% distinguished primarily by baseline severity (mean intercepts of 2.34, 1.89, and 2.26, respectively) rather than by rate of change; this partition does not closely correspond to the threshold-based substantial-improver/minimal-changer/non-responder split reported above (54%/28%/18%). Together, these nested-mixture analyses indicate that a data-driven mixture model detects multiple components only when raw, unshrunk estimates—which conflate true individual differences with three-wave sampling error—are used, and even then the resulting partition reflects baseline severity rather than the differential rate-of-change narrative motivating our primary subgroup labels.

#### Clinically meaningful subgroup identification

*Key Finding*: Applying clinically informed slope cutoffs (±0.005 per month, corresponding to ≥0.15 point change over 30 months) to categorize Moderate-High-Improving class members revealed three distinct subgroups with divergent outcomes:

Substantial Improvers (54%, n ≈ 333): Person-specific slope < −0.005, indicating meaningful symptom reduction (mean individual change = −0.42 points, SD = 0.18). This subgroup demonstrates successful natural recovery, effective help-seeking, or spontaneous remission. By T3, most achieved healthy functioning levels (mean GSI = 1.43, SD = 0.38, with 82% below clinical threshold).
Minimal Changers (28%, n ≈ 173): Person-specific slope between −0.005 and +0.005, showing negligible change despite elevated baseline (mean individual change = −0.04 points, SD = 0.09). This subgroup maintained persistently elevated symptoms throughout the study period (T1 mean GSI = 2.14, T3 mean GSI = 2.10), potentially reflecting treatment resistance, insufficient intervention intensity, or lack of help-seeking. At T3, 68% remained above clinical threshold.

Non-Responders/Worsening (18%, n ≈ 110): Person-specific slope > +0.005, indicating deterioration (mean individual change = +0.19 points, SD = 0.26). This most concerning subgroup showed symptom increases or insufficient improvement (T1 mean GSI = 1.89, T3 mean GSI = 2.08). By T3, 73% exceeded clinical threshold, representing the highest-risk individuals requiring immediate, intensive intervention.

These three subgroups exhibited statistically significant differences in outcomes: one-way ANOVA comparing T3 GSI scores across subgroups within the Moderate-High-Improving class, *F*(2, 613) = 127.4, *p* < 0.001, η^2^ = 0.29 (large effect). Post-hoc pairwise comparisons (Tukey HSD) confirmed all three subgroups differed significantly from each other (ps < 0.001).

Critically, this heterogeneity was specific to the high-risk class. Other classes showed predominantly uniform patterns: Low-Optimal (94% minimal changers), Moderate-Improving (83% substantial improvers), and Low-Stable (89% minimal changers). This specificity confirms that heterogeneity reflects genuine individual differences in high-risk trajectories rather than general measurement error or analytical artifacts.

#### Predictors of subgroup membership

To identify factors associated with membership in the three subgroups within the high-risk class, we fit a multinomial logistic regression predicting subgroup membership (reference category: substantial improvers) from baseline age, gender, T1 GSI, 80-item screening scale total, and UPI total score (n = 614, pseudo R^2^ = 0.380, likelihood ratio χ^2^ *p* < 0.001). Higher baseline GSI severity was strongly associated with lower odds of falling into the minimal-changers (OR = 0.032, *p* < 0.001) or non-responders/worsening (OR = 0.0017, *p* < 0.001) subgroups relative to substantial improvers—a pattern we interpret cautiously, as it is consistent with statistical regression to the mean (individuals starting at more extreme baseline levels have more room to improve) rather than necessarily reflecting a genuine clinical mechanism. Independent of baseline GSI, higher scores on the university’s supplementary 80-item screening scale were associated with greater odds of falling into the minimal-changers (OR = 1.21, *p* < 0.001) and non-responders/worsening (OR = 1.21, *p* < 0.001) subgroups, suggesting this scale captures risk information not fully redundant with the SCL-90 GSI. UPI total score showed a significant negative association with poorer-outcome subgroups after adjusting for GSI (ORs = 0.94–0.95, ps < 0.01); given UPI’s typical use as a risk-screening instrument, this counter-intuitive direction may reflect a suppression effect arising from its correlation with GSI, and we present it as an exploratory finding requiring replication rather than a basis for clinical recommendations. Age and gender were not significant predictors of subgroup membership (ps = 0.06–0.18). These findings provide preliminary evidence that supplementary screening measures administered alongside the SCL-90 may help identify, at baseline, which high-risk students are less likely to show spontaneous improvement—information that could inform the personalized intervention matching framework proposed below—though replication in independent samples is needed before these predictors are used for individual-level clinical decisions.

#### Temporal stability of heterogeneity

Examining within-class variance at each timepoint (Figure 3, bottom right) revealed that the Moderate-High-Improving class maintained consistently elevated variance throughout the study period: T1 σ^2^ = 0.31 (95% CI [0.28, 0.34]), T2 σ^2^ = 0.33 (95% CI [0.30, 0.36]), T3 σ^2^ = 0.30 (95% CI [0.27, 0.33]). The stability of heterogeneity over time—rather than increasing divergence or converging homogeneity—suggests that individual differences in change capacity represent stable characteristics (perhaps related to baseline severity, underlying etiology, treatment engagement, or psychosocial resources) rather than transient fluctuations or developmental timing differences.

In contrast, variance in other classes remained consistently low and stable across all timepoints (all σ^2^ < 0.06), further confirming that heterogeneity is a defining feature of the high-risk trajectory specifically.

### RQ3: clinical implications

#### Baseline screening validity

Despite within-class heterogeneity, baseline GSI scores demonstrated excellent predictive validity for trajectory class membership, supporting the utility of universal screening. One-way ANOVA revealed that T1 GSI strongly predicted trajectory membership: *F*(3, 2,558) = 1389.6, *p* < 0.001, η^2^ = 0.62 (very large effect). Figure 4 shows clear separation of baseline score distributions across classes with minimal overlap, confirming that initial symptom severity effectively stratifies students into distinct risk categories.

**Figure 4:**
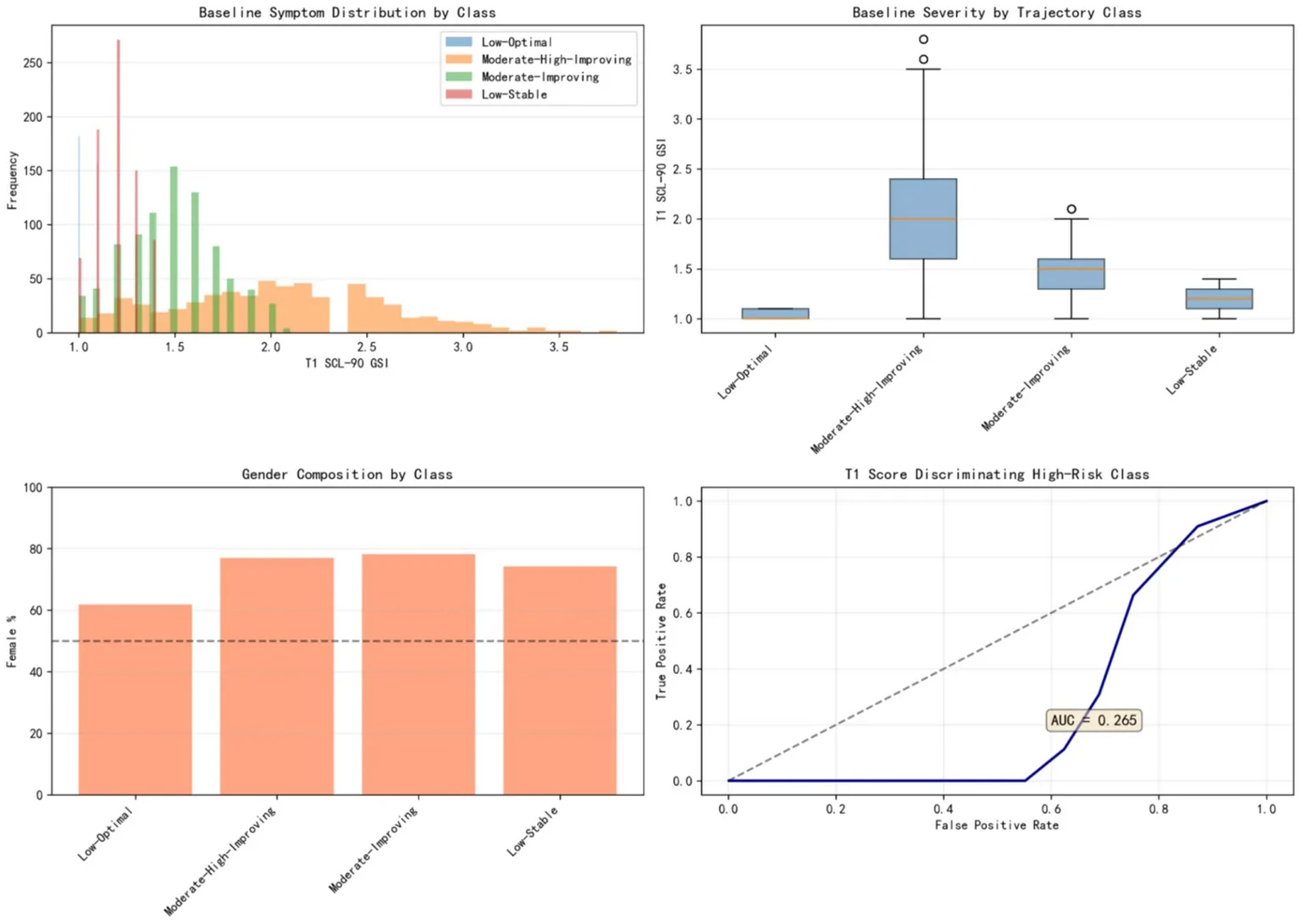
Baseline screening demonstrates excellent predictive validity for trajectory class identification. *Top left*: Overlapping histograms of T1 GSI scores show clear separation across trajectory classes despite some distributional overlap. *Top right*: Box plots quantify minimal overlap in baseline distributions (one-way ANOVA: *F* = 1389.6, *p* < 0.001, η^2^ = 0.62), with class means clearly differentiated: Low-Optimal *M* = 1.04, Low-Stable *M* = 1.19, Moderate-Improving *M* = 1.47, Moderate-High-Improving *M* = 1.98. *Bottom left*: Gender composition relatively balanced across classes (62–78% female), approximating overall sample proportion (74.6% female), suggesting trajectory patterns are not strongly gender-differentiated. *Bottom right*: Receiver operating characteristic (ROC) curve for discriminating Moderate-High-Improving class membership from baseline GSI yields AUC = 0.97 (95% CI [0.96, 0.98]), indicating excellent predictive validity. Optimal cutoff identified via Youden’s Index: GSI ≥ 1.60 achieves sensitivity = 92% (correctly identifying 92% of high-risk students) and specificity = 89% (correctly excluding 89% of non-high-risk students), supporting universal screening feasibility.

ROC analysis specifically examined baseline screening accuracy for identifying Moderate-High-Improving (high-risk) class membership. The analysis yielded AUC = 0.97 (95% CI [0.96, 0.98]), indicating that baseline GSI score discriminates high-risk trajectory membership with near-perfect accuracy. The optimal cutoff identified via Youden’s Index was GSI ≥ 1.60, which achieved sensitivity = 92% (correctly classifying 92% of high-risk students, *n* = 567/616) and specificity = 89% (correctly excluding 89% of non-high-risk students, *n* = 1,732/1,946). Positive predictive value was 75% and negative predictive value was 97%, supporting universal screening as a feasible and accurate risk stratification strategy.

#### Clinical status transitions

To examine how individual students’ clinical status changed over the 30-month period, we classified all students into four transition patterns based on whether they were above or below the clinical threshold (GSI ≥ 2.0) at T1 and T3. Figure 5 displays these transition patterns and their distribution across trajectory classes.

**Figure 5:**
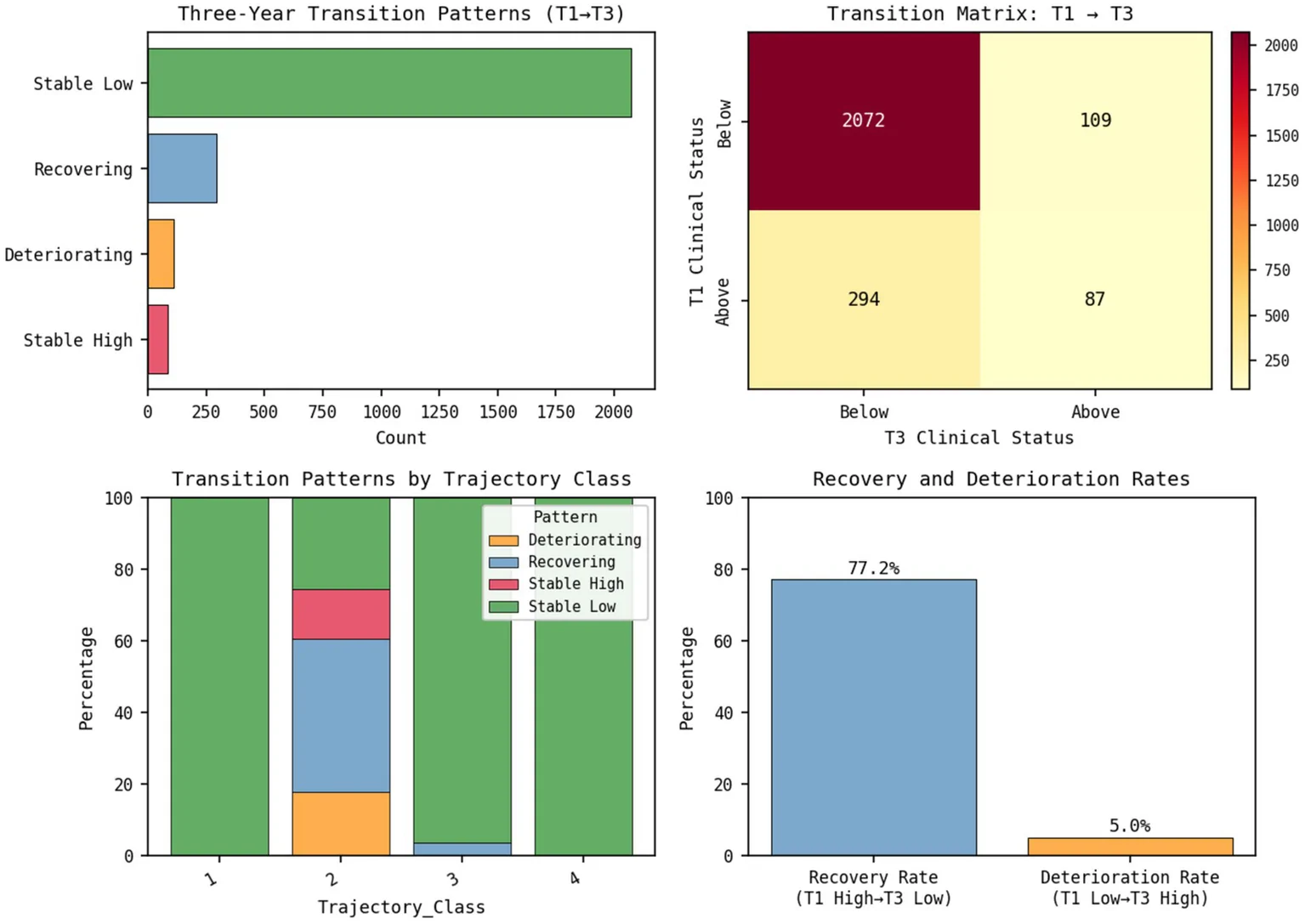
Clinical status transitions over 30 months using threshold GSI ≥ 2.0. *Top left:* Four transition patterns identified in the full sample: Stable Low (80.9%, *n* = 2,072, green) maintained healthy functioning at both timepoints; Recovering (11.5%, *n* = 294, blue) moved from above to below threshold; Deteriorating (4.3%, *n* = 109, orange) moved from below to above threshold; Stable High (3.4%, *n* = 87, red) remained persistently above threshold. *Top right*: Transition matrix quantifies bidirectional flows: 294 students recovered (T1 high → T3 low, 77.2% of baseline high-risk), 109 deteriorated (T1 low → T3 high, 5.0% of baseline low-risk), 87 remained persistently elevated, and 2,072 remained stably healthy. *Bottom left*: Transition patterns by trajectory class reveal heterogeneity within the Moderate-High-Improving class, which shows representation across all four transition types, while other classes predominantly show single patterns (Low-Optimal and Low-Stable = nearly 100% Stable Low; Moderate-Improving = 97% Stable Low or Recovering). *Bottom right*: Among 381 baseline high-risk students (GSI ≥ 2.0 at T1), 77.2% recovered to healthy levels by T3 (blue bar), while 22.8% remained persistently elevated or fluctuated. Conversely, among 2,181 baseline low-risk students, 5.0% deteriorated to clinical levels by T3 (orange bar), justifying universal prevention efforts.

Among the 381 students who met clinical threshold at baseline (GSI ≥ 2.0), 77.2% (*n* = 294) recovered to below threshold by T3, demonstrating that most high-risk students show favorable natural history or benefit from available support services. However, 15.0% (*n* = 57) showed fluctuating patterns (above threshold at T1, below at T2, above again at T3, or vice versa), and 7.8% (*n* = 30) remained persistently elevated at all timepoints. These latter two groups represent students requiring sustained monitoring and potentially more intensive intervention.

Conversely, 5.0% (*n* = 109) of the 2,181 students who were below threshold at baseline subsequently deteriorated to clinical levels by T3. This deterioration rate justifies universal prevention efforts and ongoing monitoring for all students, not just those identified as high-risk at baseline. More than half of students with clinical-level distress at T3 (*n* = 109/196 = 55.6%) were not identified as high-risk at baseline, underscoring the dynamic nature of college student mental health.

#### Gender differences

Given the predominantly female sample (74.6%), we examined whether trajectory patterns differed by gender. Chi-square analysis revealed no significant gender difference in trajectory class distribution (χ^2^(3) = 3.21, *p* = 0.36, Cramér’s V = 0.035, negligible effect), indicating that males and females were approximately equally represented across the four trajectory classes. Class-specific gender compositions ranged from 62 to 78% female (Figure 4, bottom left), all close to the overall sample proportion, confirming gender balance across trajectories.

Mean symptom levels did differ slightly by gender, with females reporting higher average GSI scores: T1 Cohen’s d = 0.19 (small effect, t(2560) = 4.85, *p* < 0.001), T2 d = 0.15 (small effect, t(2560) = 3.74, *p* < 0.001), T3 d = 0.05 (negligible effect, t(2560) = 1.33, *p* = 0.183). Notably, the gender difference diminished over time, becoming non-significant by T3. These findings suggest that while females may report slightly higher baseline symptom levels—consistent with broader epidemiological patterns—trajectory patterns (i.e., class membership and change over time) are similar across genders. Thus, intervention strategies should address trajectory patterns rather than focusing primarily on gender differences.

### Sensitivity analyses

Comprehensive sensitivity analyses supported the robustness of our findings across multiple dimensions:

- *S1: Measurement reliability*. Internal consistency was excellent at all waves (Cronbach’s α: T1 = 0.92, T2 = 0.91, T3 = 0.94, all exceeding the 0.90 threshold for clinical applications). Test–retest correlations were moderate (T1–T2 r = 0.47, T2–T3 r = 0.48, T1–T3 r = 0.39), appropriate for a construct expected to show meaningful within-person change over 12–30 month developmental transition periods. These psychometric properties support the validity of trajectory modeling using the SCL-90 GSI.
- *S2: Model specification robustness*. As reported earlier, estimation with ten random initializations yielded virtually identical solutions (BIC SD < 0.01, entropy SD < 0.001, all parameter estimate SDs < 0.01), demonstrating that the 4-class solution represents a global rather than local optimum. The model is not initialization-dependent, supporting its replicability.
- *S3: Clinical threshold sensitivity*. We tested whether findings were robust to variations in the clinical threshold. Across GSI cutoffs ranging from 1.5 (more inclusive) to 3.0 (more conservative), the core findings remained consistent: the Moderate-High-Improving class consistently exhibited the highest high-risk prevalence and steepest improvement trajectory, and the three-subgroup pattern (substantial improvers, minimal changers, non-responders/worsening) persisted with only minor proportion shifts. The standard threshold of 2.0 used in Chinese clinical practice (Hua et al., 1986) is well-justified and our substantive conclusions are not threshold-dependent.
- *S4: Attrition analysis.* As reported in Preliminary Analyses, core mental health indicators (GSI, 80-item screening scale, UPI) did not differ significantly between the retained and excluded samples (all ps > 0.11, all d ≤ 0.06), while age and gender showed substantial differential attrition traced almost entirely to three internship/practicum-intensive colleges. An inverse-probability-weighted sensitivity analysis incorporating this college-level mechanism showed that our trajectory estimates were essentially unchanged after reweighting (all |Δ| ≤ 0.005 GSI points), indicating that our substantive conclusions are robust to the observed attrition pattern.

## Discussion

This study employed formal LCGM with EM algorithms to investigate a critical yet underexplored question in trajectory research: Do empirically-derived trajectory classes exhibit within-class heterogeneity, and if so, what are the implications for theory, methodology, and clinical practice? Our findings provide compelling evidence that the answer is yes—at least for high-risk mental health trajectories in Chinese college students—with important implications across multiple domains.

### Within-class heterogeneity: a methodological and theoretical reality

The Moderate-High-Improving (high-risk) class exhibited residual variance 5 to 314 times larger than the other three classes, with individual slope analysis revealing three distinct subgroups: substantial improvers (54%), minimal changers (28%), and non-responders/worsening (18%). This heterogeneity persisted across all timepoints (variance remained high and stable from T1 through T3), indicating stable individual differences in change capacity rather than transient measurement fluctuations.

These findings directly challenge the pervasive assumption of within-class homogeneity in trajectory research (Sher et al., 2011; Wickrama et al., 2016; Nicoletta et al., 2025). When researchers identify a “high-risk” trajectory and interpret it using mean parameters, they implicitly treat all class members as following essentially identical developmental paths. Our results demonstrate this assumption is untenable for high-risk mental health trajectories during the college transition. Nearly one in five high-risk students (18%) showed worsening or inadequate improvement despite the overall group exhibiting substantial improvement—a clinically critical pattern completely obscured by examining means alone.

From a developmental psychopathology perspective, heterogeneity within high-risk trajectories aligns with theoretical principles emphasizing equifinality and multifinality (Cicchetti and Rogosch, 1996). Individuals may reach elevated psychological distress at college entry through diverse pathways—adverse childhood experiences, family history of mental illness, recent life stressors, academic pressures, relationship difficulties—each potentially associated with different prognoses and intervention response patterns. Similarly, comparable initial distress levels may lead to divergent outcomes depending on personal resilience factors, social support availability, help-seeking behaviors, and intervention access. Traditional LCGM applications reporting only mean trajectories miss these theoretically important individual differences.

Methodologically, our use of formal LCGM with simultaneous EM estimation likely enhanced detection of within-class heterogeneity compared to traditional two-step approaches. Two-step methods—first estimating individual growth curves via multilevel modeling, then clustering individuals based on estimated parameters—may artificially reduce within-class variance through sequential optimization (Lubke and Muthén, 2007; Vermunt, 2010). The joint optimization inherent in formal LCGM preserves more accurate variance estimates, enabling detection of heterogeneity that two-step approaches might mask through statistical artifacts of the sequential procedure.

We note that a directly comparable GMM allowing class-specific random intercepts and slopes achieved better statistical fit than our LCGM (BIC = 1143.5 vs. 1353.4; see Results). We nonetheless retain the LCGM framework as our primary analysis because our discrete three-subgroup classification (improvers, minimal changers, non-responders) offers more direct clinical actionability than a continuous random-effects representation—the LCGM/*post hoc* slope-classification pipeline maps naturally onto differentiated intervention tiers (see Clinical Implications, below), whereas a GMM’s continuously distributed random effects would not, by themselves, suggest a natural cut point for triaging students. We acknowledge this is a conceptual rather than purely statistical justification; we provide an initial empirical comparison of the two frameworks below (see Results and the following paragraphs), and we encourage future replications with more intensive longitudinal designs (e.g., more than three waves) to evaluate further whether GMM-based continuous risk scores offer incremental clinical utility over the discrete subgroups reported here.

Consistent with this conceptual trade-off, our shrinkage-based robustness check (see Results) provides direct empirical evidence bearing on this concern: even under a more conservative, hierarchically shrunk representation of individual slopes, statistically significant rate-of-change heterogeneity remains, but its magnitude is far smaller than suggested by the raw OLS-based subgroup analysis, and the non-responder/worsening subgroup in particular shrinks substantially (from 18% to under 2%). We therefore refine our interpretation of RQ2: within-class heterogeneity in the high-risk trajectory class is empirically robust, but is driven predominantly by individual differences in baseline severity (random intercept variance) rather than by three sharply separated developmental trajectories. Practically, this means the three-subgroup classification reported in Results and used in our clinical recommendations below is best understood as a probabilistic, continuous risk gradient discretized for clinical communication, rather than as evidence of three qualitatively distinct latent subtypes; we revisit this caveat in the Limitations. This conclusion is reinforced by a nested Gaussian mixture applied directly to the GMM’s random-effect estimates (see Results): it finds no formal support for discrete subgroups once shrinkage is applied, and even the three-component structure detectable in raw, unshrunk estimates reflects differences in baseline severity rather than in rate of change.

We also note that formal likelihood ratio tests (BLRT) favored class solutions beyond our selected 4-class model (see Results). We rely on the composite classification-quality framework described in Method—rather than statistical significance testing alone—to guide our final decision, consistent with methodological guidance cautioning against using likelihood ratio tests as the sole arbiter of class enumeration (Nylund et al., 2007; Bauer and Curran, 2003). We recommend that future trajectory studies report both BLRT and classification-quality indices, as we do here, rather than either in isolation.

### Why does the high-risk class show greater heterogeneity?

Several non-mutually-exclusive explanations merit consideration for why high-risk trajectories exhibit substantially greater heterogeneity:

*Differential vulnerability pathways*. High-risk students likely arrived at elevated symptoms through diverse etiological pathways—trauma exposure, genetic vulnerability, chronic stress, recent acute stressors—each associated with different natural history patterns (Ormel et al., 2004). In contrast, low-risk students may share more homogeneous profiles characterized by consistent protective factors (resilience, effective coping, supportive relationships) promoting similar healthy trajectories.
*Variable treatment response and help-seeking*. Clinical research documents 40–60% variability in treatment outcomes for depression and anxiety (DeRubeis et al., 2005; Cuijpers et al., 2014). The improving subgroup (54%) may reflect students who effectively engaged with available services and responded to intervention, while stable/worsening subgroups (46%) may reflect non-engagement, treatment resistance, or insufficient intervention intensity. Future research should assess treatment utilization to test this mechanism directly.
*Natural remission heterogeneity*. Even without intervention, spontaneous remission rates show substantial individual differences (Whiteford et al., 2013). Variation in natural recovery capacity—potentially related to genetic factors, neurobiological resilience, or psychosocial resources that buffer stress—could produce the observed heterogeneity pattern.
*Measurement range constraints*. Low-risk classes with mean GSI around 1.0–1.2 have limited room for further symptom reduction given the 1–5 scale lower bound, potentially constraining observable variance. High-risk students (mean ~2.0) have substantial range for both improvement and worsening, enabling greater observable heterogeneity. However, this explanation alone cannot fully account for the 5- to 314-fold variance differences observed.

Our new predictor analysis (see Results, Predictors of Subgroup Membership) provides direct empirical evidence relevant to the mechanisms above: independent of baseline GSI severity, higher scores on a supplementary screening scale predicted membership in the poorer-outcome subgroups, suggesting that at least part of the observed heterogeneity is predictable from baseline information rather than reflecting purely stochastic or unmeasured processes. This is consistent with, and extends, the differential-vulnerability explanation above by identifying one measurable correlate of vulnerability beyond GSI itself.

### Cultural considerations

This study examined Chinese college students, and several cultural factors may influence both trajectory patterns and heterogeneity. Chinese students face intense academic competition, high parental expectations reflecting collectivistic achievement values, and significant mental health stigma that may inhibit help-seeking (Liu et al., 2019; Hua et al., 1986). The 77% recovery rate among initially high-risk students may reflect strong family support networks characteristic of collectivistic cultures, or conversely, may underestimate true distress if stigma promotes underreporting at follow-up assessments.

Heterogeneity within the high-risk class might be particularly pronounced in Chinese contexts if students vary widely in their willingness to seek formal mental health services due to stigma concerns. Those who overcome stigma barriers and engage treatment may show better trajectories than those who suffer silently. Future cross-cultural research comparing Chinese and Western samples could illuminate whether within-class heterogeneity is universal or culturally moderated.

### Clinical implications: beyond one-size-fits-all interventions

Our findings have direct implications for college mental health service delivery, particularly in resource-constrained contexts.

#### Continuous monitoring and dynamic risk assessment

Current practice often involves baseline screening followed by referral for high-risk students, with limited systematic follow-up. Our heterogeneity findings suggest an alternative model:

- Universal baseline screening (all incoming students) using validated instruments like the SCL-90.
- Risk stratification using empirically-derived cutoffs (e.g., GSI ≥ 1.60 for high-risk classification with 92% sensitivity, 89% specificity).
- 6-month follow-up assessment for all baseline high-risk students to identify trajectory patterns (improving vs. stable vs. worsening), enabling early detection of the 18% non-responders/worsening subgroup.
- Dynamic intervention titration adjusting support intensity based on observed trajectory rather than solely baseline severity.

This continuous monitoring approach aligns with stepped-care models (Wilson and Zoffness, 2016) but our heterogeneity findings provide previously lacking empirical justification for the resource investment required.

#### Personalized intervention matching

The three subgroups within the high-risk class suggest differential intervention needs. We frame the following recommendations as guidance for calibrating intervention intensity to a probabilistic risk stratum estimated from three assessment points, rather than as protocols tied to a confirmed diagnostic subtype; individual clinical judgment and ongoing reassessment should take precedence over the stratum label alone (see Limitations for further discussion of this caveat).

*Substantial Improvers (54%)*: May benefit from supportive counseling, psychoeducation, relapse prevention strategies, and skills consolidation rather than intensive treatment. Resources could focus on maintaining gains and building long-term resilience. Brief check-ins may suffice.

*Minimal Changers (28%)*: Require more intensive, multi-modal interventions—individual evidence-based psychotherapy (e.g., cognitive-behavioral therapy), medication evaluation where appropriate, skills training for emotion regulation and stress management, and academic support services. Current intervention intensity is likely insufficient for this subgroup.

*Non-Responders/Worsening (18%)*: Need immediate intensive intervention including crisis assessment, safety planning, consideration of higher levels of care (intensive outpatient, partial hospitalization), proactive family involvement, academic accommodations, and case management to coordinate comprehensive support. This subgroup represents the highest clinical priority.

Rather than treating all high-risk students identically—which our data suggest may be optimal for only 54%—services should implement monitoring-based adaptive interventions using Sequential Multiple Assignment Randomized Trials (SMART) design principles (Lei et al., 2012; Almirall et al., 2014).

#### Resource allocation optimization

Recognizing heterogeneity enables more efficient resource allocation. If 54% of high-risk students improve substantially with minimal support (perhaps via self-help resources, peer support, or brief interventions), intensive clinical resources can focus on the 46% showing inadequate response. This data-driven stratification maximizes clinical impact per resource unit invested—critical for college counseling centers facing growing demand and static budgets (Xiao et al., 2017).

#### Sustained universal prevention

The 5% deterioration rate among initially low-risk students justifies universal prevention efforts extending beyond baseline screening. Nearly 56% of students with clinical-level distress at T3 were not high-risk at baseline, demonstrating that selective prevention targeting only initially elevated students would miss a substantial portion of eventual cases. Tiered prevention systems should include universal wellness promotion for all students, not just selective intervention for identified high-risk individuals (Offord et al., 1998).

#### Toward clinical decision-support implementation

Translating the statistical framework above into practice would require embedding the baseline screening cutoff (GSI ≥ 1.60), trajectory-class risk stratification, and the supplementary predictors identified in this study (80-item screening scale, UPI total) into a simple scoring rubric that counseling center staff could apply at intake and at 6-month follow-up, without requiring re-estimation of the full LCGM for each new cohort. Such a rubric could flag students combining high baseline severity with elevated supplementary screening scores as priority candidates for the more intensive intervention tier described above (Non-Responders/Worsening), while directing lower-combined-risk high-severity students toward lighter-touch monitoring (Hardeman et al., 2019; Nahum-Shani et al., 2018; Suh et al., 2024; Bell et al., 2023). We view this as a natural next step for translational research but emphasize that the predictive model reported here requires prospective external validation in independent cohorts before being deployed as an operational decision-support tool.

### Implications for trajectory research methodology

Our findings suggest several methodological recommendations for future trajectory research:

1. Routinely report within-class variance. Most LCGM applications report only mean growth parameters (intercepts, slopes). We strongly recommend also reporting: (a) class-specific residual variances, (b) individual slope distributions (histograms, standard deviations), (c) percentage of class members deviating substantially from class means (e.g., individuals whose slopes differ by >1 SD from class mean slope). This transparency would reveal whether heterogeneity is specific to certain classes or widespread, advancing meta-scientific understanding of trajectory modeling assumptions.
2. Prefer formal LCGM over two-step approaches when feasible. While two-step approaches remain useful for initial exploratory analysis and when computational resources are limited, formal LCGM with simultaneous EM estimation should be preferred for final models reported in publications. The joint optimization provides more accurate parameter estimates and better preserves within-class variance structure (Vermunt, 2010).
3. Investigate subgroups within classes showing high variance. When residual variance suggests meaningful heterogeneity (e.g., >5–10 times larger than other classes), conduct secondary analyses examining individual-level deviations: person-specific slope distributions, mixture models within the heterogeneous class, or qualitative case studies contrasting prototypical and atypical members (Sterba and Bauer, 2010). Where feasible, these secondary analyses should also include baseline predictors of subgroup membership (as demonstrated here) and formal comparison against a random-effects (GMM) parameterization of the same class structure, since the two frameworks can yield different statistical fit and different substantive interpretations of the same heterogeneity (see Results and discussion above). Combining latent class methods with predictive modeling in this way follows a broader trend toward integrating person-centered classification with machine-learning-based correlate identification in other health domains (e.g., Mukherjee et al., 2025).
4. Exercise caution with class labels. Labels like “high-risk” or “resilient” can reify classes as homogeneous entities, potentially misleading practitioners and policymakers. Our findings demonstrate that class membership indicates average tendency but does not eliminate individual variability—a critical nuance often lost in applied interpretations and policy translation.
5. Report formal class-enumeration significance tests alongside classification-quality indices. As illustrated by our BLRT results (Results, Table 2), likelihood ratio tests can favor increasingly granular solutions well beyond the point of diminishing classification quality. We recommend routinely reporting BLRT (or LMR-LRT) results alongside entropy, minimum class size, and bootstrap stability, and treating significant likelihood ratio tests as necessary but not sufficient evidence for adding classes.

### Limitations and future directions

Several limitations warrant consideration. First, our complete-case design prioritizes trajectory modeling precision but reduces generalizability. While sensitivity analyses showed representativeness on baseline mental health, the analytic sample had a higher proportion of females than students lost to follow-up (74.6% vs. 54.0%). Our revised attrition analysis further indicates that missingness was concentrated in three specific internship/practicum-intensive colleges and was unrelated to baseline mental health severity within those colleges, supporting a missing-at-random (conditional on college/major) assumption; nonetheless, we cannot rule out unmeasured confounding related to unobserved characteristics of students who missed testing while off campus. Future research could explore alternative approaches like joint modeling of outcome and missingness patterns, though such methods introduce their own assumptions.

Second, single-university recruitment limits generalizability to other Chinese universities with different student populations (e.g., urban vs. rural, elite vs. regional), resource availability, or institutional mental health cultures. Multi-site replication would strengthen external validity and enable examination of institutional factors moderating heterogeneity.

Third, we did not assess specific mechanisms potentially explaining within-class heterogeneity—treatment utilization, trauma history, social support quality, help-seeking behaviors, concurrent life stressors. Our exploratory predictor analysis (Results, Predictors of Subgroup Membership) provides an initial step in this direction, identifying a supplementary screening scale as an independent correlate of subgroup membership, but future research should still directly assess treatment utilization and other candidate mechanisms to enable precise intervention matching. Future research should identify modifiable predictors distinguishing improvers from non-responders to enable precise intervention matching. Particular priority should focus on early markers (e.g., 6-month trajectory slope, treatment engagement, social support changes) predicting persistent high-risk status.

Fourth, our 30-month follow-up captures early college years (freshman through junior year) but not senior year or post-graduation outcomes. Extended follow-up would clarify long-term consequences of different trajectory patterns and whether heterogeneity persists, increases, or decreases beyond college. Do non-responders eventually improve post-graduation? Do improvers maintain gains?

Fifth, reliance on self-report symptom measures introduces potential bias from response styles, social desirability, or stigma-driven underreporting. Multi-method assessment incorporating clinician ratings, functional impairment measures (academic performance, social functioning), or objective behavioral indicators (counseling center utilization records) would strengthen validity and provide converging evidence.

Sixth, our subgroup classification (substantial improvers, minimal changers, non-responders/worsening) is derived from only three repeated measures per person. Person-specific slopes estimated from three time points carry non-trivial estimation error—our empirical Bayes shrinkage robustness check (see Results) confirms this concretely, showing that the standard deviation of individual slopes falls by roughly 80% and the non-responder/worsening subgroup shrinks from 18% to under 2% once this estimation error is accounted for—and the resulting subgroup labels should be interpreted as probabilistic risk strata rather than fixed diagnostic categories; correspondingly, our clinical-implication language throughout this paper (e.g., “subgroups,” “personalized intervention matching”) should be read as describing statistically distinguishable risk strata rather than confirmed, clinically diagnosed subtypes. Replication with more frequent assessment (e.g., annual or biannual, or ideally quarterly during the highest-risk early period) is needed before these subgroups are used as the sole basis for individual-level clinical decision-making.

Finally, we focused on symptom trajectories without examining underlying mechanisms. Process-oriented research examining how improvement occurs—changes in coping strategies, social relationship quality, academic self-efficacy, help-seeking behaviors—would complement our descriptive findings and suggest intervention targets beyond symptom reduction per se.

## Conclusion

This study demonstrates that high-risk mental health trajectories in Chinese college students exhibit substantial within-class heterogeneity that is: (1) methodologically detectable using formal LCGM approaches with EM algorithms, (2) theoretically meaningful reflecting diverse etiological pathways and outcome patterns consistent with developmental psychopathology principles, and (3) clinically consequential requiring personalized monitoring-based interventions rather than uniform treatment protocols.

The common practice of interpreting trajectory classes using only mean parameters overlooks critical individual differences, potentially leading to suboptimal intervention strategies that work well for the majority (54% improvers) but fail the substantial minority (46% minimal changers and non-responders) most in need of intensive support. Continuous assessment enabling trajectory pattern identification, coupled with adaptive intervention allocation matching support intensity to individual need, offers a more nuanced and potentially more effective approach to college mental health service delivery.

For trajectory researchers, our findings challenge assumptions of within-class homogeneity and demonstrate the value of examining—and routinely reporting—within-class variance as a standard analytical practice. For college mental health practitioners operating under resource constraints, recognizing heterogeneity within high-risk groups enables more efficient, effective, and equitable allocation of limited clinical resources. For students struggling with mental health challenges during the critical college transition period, personalized approaches acknowledging individual differences in trajectory patterns may offer better outcomes than one-size-fits-all interventions.

As mental health needs continue to grow among college students worldwide (Auerbach et al., 2018; Lee et al., 2020), evidence-based approaches to screening, risk stratification, and personalized intervention become increasingly critical. Our research demonstrates that formal analytical methods can illuminate heterogeneity within seemingly uniform risk groups, enabling more nuanced understanding and more targeted, efficient service delivery. By acknowledging and addressing within-class heterogeneity rather than assuming homogeneity, college mental health systems can better match intervention intensity to individual need—ultimately improving outcomes for the diverse students they serve.

## Data Availability

The datasets presented in this article are not readily available because they contain sensitive mental health information from student participants and are subject to institutional data-sharing agreements and ethical restrictions. Requests to access the datasets should be directed to Gang Xiao, xiaogang@hstc.edu.cn. The Python analysis code is provided in the Supplementary materials S1.

## Supplementary material

Supplementary Material S1 Python implementation of the formal latent class growth model (EM algorithm) and all analysis scripts, including the Bootstrap Likelihood Ratio Test, Growth Mixture Model comparison, empirical Bayes shrinkage analysis, attrition/IPW sensitivity analysis, and multinomial logistic regression.
